# Fatalities caused by novel opioids: a review

**DOI:** 10.1080/20961790.2018.1460063

**Published:** 2018-05-07

**Authors:** Olaf H. Drummer

**Affiliations:** Department of Forensic Medicine, School of Public Health and Preventive Medicine, Faculty of Medicine, Nursing and Health Sciences, Monash University, Southbank, Victoria, Australia

**Keywords:** Forensic science, forensic toxicology, opioids, poisoning, illicit drugs, novel psychoactive drugs, fentanyl derivatives, mass spectrometry

## Abstract

Drugs related to morphine represent not only large range of important therapeutic applications for the relief of moderate to severe pain but also give rise to a relatively large series of novel opioids that mimic the action of this naturally occurring analgesic. Most of these are based on fentanyl structures that are much more potent, and dangerous, than fentanyl itself. This publication reviews reports of fatalities attributed to 15 novel opioids with the view to assessing mortality associated with their misuse as well as reviewing published analytical procedures that would be able to detect these and other novel opioids. These drugs include reports of deaths to acetylfentanyl, acrylfentanyl, butr(yl)fentanyl, carfentanil, 2- and 4-fluorofentanyls, 4-fluorobutyrfentanyl, 4-fluoroisobutyrfentanyl, furanylfentanyl, α- and 3-methylfentanyls, 4-methoxyfentanyl, ocfentanil, as well as AH-7921, U-47700 and MT-45. Most of these cases reporting a drug-caused death involved other drugs in addition to the opioid. No obvious minimum fatal concentration was discerned for any of the opioids for which details were provided, however, the more potent members required detection limits well under 1 ng/mL and often even well below 0.1 ng/mL requiring use of the most sensitive mass spectral detection procedures, particularly when screening specimens using a non-targeted mode. Four other novel opioids have been reported in admissions to hospitals include 4-chloroisobutryfentanyl, cyclopentylfentanyl and tetrahydrofuranfentanyl, all of which are likely to have the potential to cause death. It is also likely that other analogues will appear with time.

## Introduction

Morphine with its many of semi-synthetic and synthetic analogues including codeine, oxycodone, hydrocodone, hydromorphone, dihydrocodeine, ethylmorphine, methadone are widely used as analgesics to treat moderate to severe pain. Heroin, the diacetyl analogue of morphine has been, and continues to be, a major illicit opiate with estimates of usage of about 17 million users worldwide [1]. According to the United Nations Office of Drug Control prescription, opioids are used by a greater number (about 35 million, or 0.7% of the population). While much of this opioid usage relates to misuse of prescribed drugs, increasingly fentanyl and a number of potent analogues based on fentanyl, or other drugs acting on the opioid receptors, have been detected and have led to hospital admissions and even death [1–6]. This publication complements other recent reviews covering opioids and prevalence of sudden death [2,3].

There have been hundreds of deaths from fentanyl since deaths from its abuse were first reported over 30 years ago. These include those first reported in California [5], to other states including Illinois [6,7], Michigan [8,9], Florida [10], Kansas [11], Maryland [12], Massachusetts [13], Minnesota [14], New Mexico [15], and also in other parts of the world including clusters in Canada [16] and Sweden [17]. In 2015, the USA Centre for Disease Control (CDC) reported 33 091 opioid deaths, of which almost 10 000 were due to synthetic opioids other than methadone (including fentanyl and related drugs), an increase of 72% over 2014 [18].

Fentanyl is about 100 times more potent on the mu-opioid receptor than morphine and has now been detected in batches of heroin. This is a particularly dangerous combination and has led to increased risk of sudden drug-caused death [8,19].

This publication reviews reports of these novel psychoactive opioids that have led to fatalities and provides an overview of their concentrations and circumstances in reported fatalities, and the detectability of these drugs in biological specimens.

## Methods

Publications in the English language that reported fatalities from use of a fentanyl derivative or any other novel opioid were searched in PubMed as well as Scopus. Publications not captured in the initial searches but cited in publications were also retrieved and included, where relevant. Published methods were included in this publication where the procedure was targeted to measuring a number of fentanyls and other novel opioids or a number of fentanyls and novel opioids were included in a wider analytical procedure designed for blood and/or urine.

## Results

### Structural characteristics

Synthetic opioids related to the phenylheptylamines and phenylpiperidines show significant differences in their apparent 2-D structures, although they do bind to the opioid receptors, particularly the mu-subtype, sometimes with higher affinity than morphine itself. Examples include methadone, pethidine (meperidine), propoxyphene, fentanyl and l-α-acetylmethadol (LAAM).

**Figure 1.**
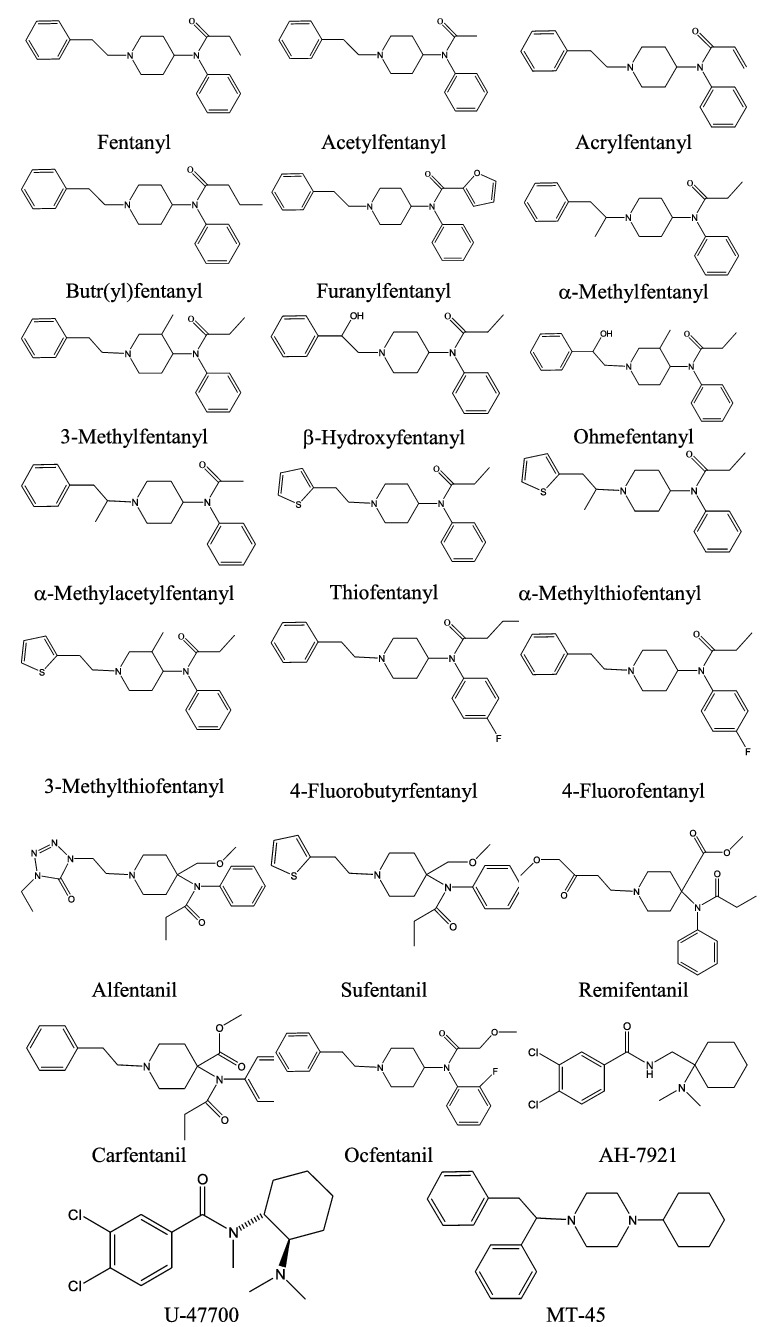
Structures of selected opioids.

Fentanyl is a piperidinyl derivative with moieties on the nitrogen and the 4-position. Alfentanil, remifentanil and sufentanil are short acting analogues of fentanyl (with piperidinyl ring) and have been approved for use in humans for some time for induction of anaesthesia (Figure 1).

The original (designer) fentanyl derivatives included α-methylfentanyl and 3-methylfentanyl [20]. China White, or 3-methylfentanyl, is some 6 000 times more potent than morphine and is active from a few micrograms. They are fentanyl analogues, e.g. a methyl substitution on fentanyl. More recently, replacement of the propionyl moiety of fentanyl with acetyl (acetylfentanyl), acryl (acrylfentanyl), butyryl (butyrfentanyl) or furanyl (furanylfentanyl) has led to a series of novel opioids (Figure 1). Other variations of the fentanyl molecule include replacement of the phenethyl moiety with β-hydroxy substituted form (with and without other substitutions) (β-hydroxyfentanyl, ohmefentanyl, 2-thiophene-ethyl, thiofentanyl, 3-methylfentanyl, α-methylthiofentanyl), and substitutions on the *N*-phenyl ring, often with 4-flouro (4-fluorofentanyl, 4-fluorobutyrfentanyl, 4-fluoroisobutyrfentanyl).

Carfentanil, with modifications at the 4-piperidinyl end is possibly the most potent commercially available fentanyl derivative that also has a veterinary use for analgesia in large animals [21].

Newer novel opioids that have more structural variations to the fentanyl structure and have been detected in recent opioid-caused deaths include AH-7921 and the isomer U-47700, and MT-45 (Figure 1).

A more detailed review of the structure-activity relationships of a larger series of fentanyl analogues can be seen in the following publications [22,23].

### Fentanyl fatalities

Table 1 summarizes the papers that describe fatalities arising from novel opioids including a sampling of those that have arisen from fentanyl itself.

**Table 1.** Publications reporting fatalities from fentanyl and other novel opioids.

Opioid(s) detected and country	Analytical method	Results	Comments	Reference*
**Selected fentanyl publications**				
Eight fentanyl fatalities in Sweden	GC-MS-SIM	Blood (f) 0.2–17 ng/g (median 5 ng/g)	All involved other drugs including 5 with alcohol	Kronstrand et al. (1997) [17]
25 fatalities from fentanyl in Los Angeles, California (USA)	GC-MS-SIM	Blood (f) 3.1–43 ng/mL (*n* = 13); blood (h) 1.8–139 ng/mL (*n* = 23); liver 5.8–613 μg/kg (*n* = 22)	Abuse of transdermal patches; 15 were accidents caused by drug misuse and often with multiple drugs	Anderson et al. (2000) [31]
19 fentanyl fatalities in south western Virginia (USA)	GC-MS-SIM	Blood 2–48 ng/mL (median 18 ng/mL)	Misuse of fentanyl or abuse of fentanyl transdermal patches. Routes of administration were transdermal, transmucosal/oral, intravenous, and combinations of routes of administration. Others drugs detected	Kuhlman et al. (2003) [33]
Fentanyl patches in Belgium	LC-MS/MS	Fentanyl/(norfentanyl): blood (f) 21 ng/mL (<2 ng/mL); blood (left ventricular) 35 ng/mL (4 ng/mL); blood (s) 28 ng/mL (3 ng/mL); VH 20 ng/mL (<2 ng/mL)	10 Durogesic patches (100 μg/h) on body (elderly woman with cancer). No other drugs detected	Coopman et al. (2007) [27]
101 fentanyl deaths in Wayne county, Michigan (USA) (2005–2006)	GC-MS-SIM	Blood (f) 4–69 ng/mL (median 20 ng/mL); blood (h) 2–51 ng/mL (median 20 ng/mL)	A number had also heroin use confirmed possibly with added fentanyl; other drugs also detected; most had pulmonary edema and many also had coronary artery disease	Algren et al. (2013) [8]
81 fentanyl deaths in Montgomery county, Ohio (USA)	GC-MS	56 cases without concomitant use of heroin or cocaine: blood 1–48 ng/mL (median 9 ng/mL); 12 cases also with concomitant heroin use: blood 1–46 ng/mL (median 12 ng/mL); 7 cases also concomitant cocaine use: blood 3.3–34 ng/mL (median 6.3 ng/mL) and 6 with concomitant heroin and cocaine use: blood 3.9–60 ng/mL (median 14.5 ng/mL)	Most had additional drugs; evidence of diversion from pharmaceutical grade fentanyl and often disguised as heroin	Marinetti et al. (2014) [28]
Large series of fentanyl fatalities in Florida (USA)	GC-MS	Blood 2.5–50 ng/mL (median 9.7 ng/mL) (*n* = 46)	Fentanyl intoxication often involving other drugs; other cases died from other causes	Lee et al. (2016) [10]
**Other fentanyl-related opioids**				
α-Methylfentanyl fatality in USA	GC-NPD	Blood 3.1 ng/mL; liver 78 ng/mg; bile 64 ng/mL	Despropionylfentanyl also detected presumptively as a possible metabolite	Gillespie et al. (1982) [62]
Sufentanil fatality in Missouri (USA)	GC-MS-SIM	Blood (h) 1.1 ng/mL; urine 1.3 ng/mL; VH 1.2 ng/mL	Young male anaesthetist self-injection; also with midazolam (Blood 50 ng/mL)	Ferslew et al. (1989) [88]
Three 3-methylfentanyl fatalities in Finland	LC-MS/MS	Cis-3-methyl fentanyl: blood 0.3–0.9 ng/mL (mean 0.5 ng/mL)	IV use; two with heroin, amphetamine and other drugs detected, 2 with liver disease; aged 30–41 years	Ojanpera et al. (2006) [64]
Series of 3-methylfentanyl deaths in Estonia	LC-MS/MS	Cis-3-methylfentayl: blood 0.06–3 ng/mL (median 0.9 ng/mL) Trans-3-methylfentanyl: blood 0.1–3.2 ng/mL (median 0.6 ng/mL)	100's in deaths in Estonia mainly from IV use, often with other drugs; site of blood not specified, average age 26 years	Ojanpera et al. (2008) [65]
14 fatalities from acetylfentanyl in USA	GC-MS	No details provided	Rhode Island (USA)	MMWR (2013) [112]
Nine AH-7921 fatalities in Sweden	HR-LC/MS	Blood (f) 0.08–0.99 μg/g (median 0.4 μg/g)	All cases involved other drugs and most victims had heavy lungs	Kronstrand et al. (2014) [68]
AH-7921 fatality in Delaware (USA)	GC-MS-SIM	Blood (f) 9.1 mg/L; blood (h) 3.9 mg/L; SC 120 mg	No other drugs were detected in blood	Vorce et al. (2014) [69]
Two AH-7921 fatalities in Norway	LC-MS/MS, HR-MS	Case 1: blood (f) 0.43 mg/L Case 2: blood (f) 0.33 mg/L	Case 1: 2-fluoroamphetamine, 3-methmethcathinone, codeine also detected in contributory concentrations Case 2: methoxetamine, etizolam, phenazepam, 7-aminonitrazepam, diazepam also detected in contributory concentrations	Karinen et al. (2014) [70]
28 MT-45 deaths in Sweden	No information given but likely to be similar to other publications reported by the Swedish laboratory	Blood 0.008–1.9 μg/g (median 0.35 μg/g)	Almost all used other drugs; two cases died from pneumonia, presumably secondary to drug toxicity and one case was an injury death, 6 cases were still under investigation	Evans-Brown et al. (2014) [75]
Five AH7921 fatalities in UK	No details	Blood 0.05–4.46 mg/L (median 0.58 mg/L)	No more details, other than additional drugs detected; two of which contributed to death.	Elliott et al. (2014) [71]
Acetylfentanyl fatality in San Diego, California (USA)	GC-MS-SIM	Blood (p) 260 ng/mL; blood (c) 250 ng/mL; VH 240 ng/mL; urine 2 600 ng/mL; liver 1 ng/mg	Young male with history of heroin abuse; likely IV use; initially detected as positive in fentanyl immunoassay	McIntyre et al. (2015) [39]
14 acetylfentanyl fatalities in Rhode Island (USA)	ELISA and GC-MS with 2 ng/mL cut-off	No blood concentration data provided	Most involved other drugs as well including cocaine, morphine/heroin, ethanol and benzodiazepines	Lozier et al. (2015) [116]
MT-45 fatality in USA	LC-MS/MS	Blood (f) 0.52 mg/L	Etizolam (0.035 mg/L), diphenhydramine 0.22 mg/L	Papsun et al. (2016) [77]
Ocfentanil death in Belgium	LC-MS/MS	Ocfentanil: blood (f) 0.015 mg/L; VH 0.012 mg/L; urine 0.006 mg/L	Young male snorting brown powder purchased over Internet. No other drugs detected in blood	Coopman et al. (2016) [66]
Butyrlfentanyl and acetyl fentanyl fatality in San Diego, California (USA)	GC-MS-SIM	Butrylfentanyl: blood (f) 0.058 mg/L; blood (c) 0.097 mg/L, liver 0.32 mg/kg; VH 0.04 mg/L; urine 0.67 mg/L; SC 170 mg Acetylfentanyl: blood (f) 0.038 mg/L; blood (c) 0.032 mg/L; liver 0.11 mg/kg; urine 0.54 mg/L; SC <70 mg	44-year old man found dead on bathroom floor: history of IV drug use; benzoylecgonine and levamisole also detected in blood	McIntyre et al. (2016) [47]
Two butyrylfentanyl fatalities in Richmond, Virginia or Tampa, Florida (USA)	LC-MS/MS	Case 1: butyrylfentanyl only detected, blood (f, h) 0.099, 0.22 mg/L; VH 0.032 mg/L, urine 0.064 mg/L; SC detected Case 2: butyrylfentanyl, blood (f,h) 0.004; 0.009 mg/L; VH 0.01 mg/L; urine 0.002 mg/L; SC detected; acetyl fentanyl, blood (f,h) 0.021, 0.095 mg/L; VH 0.068 mg/L; urine 0.008 mg/L; SC detected	Case 1: Middle-aged woman found collapsed in bathroom, but died from drug toxicity a little while later Case 2: Middle-aged woman found deceased on her bed; known to abuse oxycodone with previous suicide attempts; also alprazolam and ethanol (0.011 g/100 mL) detected	Poklis et al. (2016) [117]
U-47700 fatality in UK	LC-MS/MS, HR-MS, PAD	Blood (f) 1.46 mg/L, also *N*-desmethyl- and *N,N*-didesmethyl metabolites likely	Young male found dead at home, also snorted mirtazapine, and used cannabis, ketamine and legal highs; no disease	Elliott et al. (2016) [79]
U-47700 fatality in Belgium	LC-MS/MS	U-47700: blood 13.8 ng/mL; urine 71 ng/mL Fentanyl also detected in blood 10.9 ng/mL	Young male found dead at home inhaling fumes from a powder; sertraline 0.18 mg/L also detected	Coopman et al. (2016) [80]
Series of U-47700 and/or furanylfentanyl involved drug deaths in USA	LC-MS/MS	U-47700: blood 0.017–490 mg/L (median 0.247 mg/L) (*n* = 16) Furanylfentanyl: blood 0.002–76 mg/L (median 0.013 mg/L) (*n* = 8)	Blood, mainly femoral, some central. All bar one case involved multiple drugs including some with other opioids	Mohr et al. (2016) [56]
Acetylfentanyl fatality in West Virginia (USA)	LC-MS/MS, HR-MS	Blood (s) 235 ng/mL; liver 2 400 ng/g; urine 234 ng/mL; vitreous fluid 131 ng/mL	Young male found dead following likely IV injection; tadalafil and testosterone also detected	Cunningham et al. (2016) [89]
Acetylfentanyl, 4-methoxybutyrfentanyl and furanylfentanyl intoxications with survival in Sweden	LC-MS/MS, HR-MS	Acetylfentanyl: serum 0.6–52 ng/mL (*n* = 9) 4-Methoxybutyrfentanyl: serum 1.3–11 ng/mL (*n* = 4) Furanylfentanyl: serum 148 ng/mL (*n* = 1)	Other psychoactive drugs detected; many used nasal route, some oral. STRIDA project	Helander et al. (2016) [37]
Butyrfentanyl fatality in Switzerland	LC-MS/MS, HR-MS	Blood (f) 66 ng/mL; blood (h) 39 ng/mL; liver 57 ng/g; highest concentration detected in lung tissue; carboxybutyr-, hydroxy-, desbutyl- and norbutyl- metabolites detected	Young male with history of drug use found dead in bathroom of his apartment	Staeheli et al. (2016) [48]
Carfentanil and furanylfentanyl in Florida (USA)	GC-MS-SIM	Case 1: blood (h) Carfentanil 1.3 ng/mL; furanylfentanyl 0.34 ng/mL; fentanyl 6 ng/mL Case 2: blood (h) Carfentanil 0.12 ng/mL	No fentanyl detected in femoral blood. Trace hydromorphone, morphine (total) and 6AM also detected in case 1. Cocaine metabolites also detected in case 2	Swanson et al. (2017) [55]
4-FBF fatality in Poland	LC-MS/MS	Case 1: blood 91 ng/mL; urine 200 ng/g; liver 902 ng/g Case 2: blood 112 ng/mL; urine 414 ng/g; liver 136 ng/g	Case 1: young male found dead Case 2: young female found dead, occasional user of novel psychoactive drugs	Rojkiewicz et al. (2016) [118]
Acetylfentanyl fatality in Japan	LC-MS/MS	Blood (h) 270 ng/mL; urine and gastric contents detected	Young male found dead by insufflation; no other drugs detected	Takase et al. (2016) [40]
Acetylfentanyl fatality in Japan	GC-MS and LC-MS/MS	Blood (f) 153 ng/mL; urine 240 ng/mL; gastric contents detected	4-Methoxy PV8 also contributed to death Blood (f) 389 ng/mL; history of methamphetamine abuse	Yonemitsu et al. (2016) [119]
Two Acetylfentanyl fatalities in Oklahoma (USA)	GC-MS-SIM	Case 1: blood (f) 192 ng/mL; blood (h) 285 ng/mL; urine 3 420 ng/mL; liver 1 100 ng/g Case 2: blood (f) 255 ng/mL; blood (h) 210 ng/mL; urine 2 720 ng/mL; VH 140 ng/mL	Case 1: young male found dead in bed; fluoxetine and methoxetamine also detected Case 2: middle aged woman found dead in bed; history of seizures and prescription drug and alcohol abuse; venlafaxine, chlordiazepoxide also detected	Fort et al. (2016) [120]
Two U-47700 fatalities in Germany	LC-MS/MS	Case 1: blood (f) 525 ng/mL; blood (h) 1 347 ng/mL; urine 1 393 ng/mL; liver 4.3 ng/mg Case 2: blood (f) 819 ng/mL; blood (h) 1 043 ng/mL; urine 1 848 ng/mL; Liver 3.1 ng/mg	Case 1: diphenidine, methoxyphenidine, ibuprofen and naloxone detected Case 2: diphenhydramine and methylphenidate detected; all in therapeutic levels	Dziadosz et al. (2017) [121]
Ocfentanil fatality in Switzerland	LC-MS/MS	Blood (f) 9.1 ng/mL (fluoride); 7.5 ng/mL (heparin); blood (h) 27.9 ng/mL; urine 480 ng/mL; nasal swab 360 ng	Young male found dead; brown powder located	Dussy et al. (2016) [67]
Severn furanylfentanyl fatalities in Sweden	LC-MS/MS	Blood 0.38 – 2.74 ng/g (median 0.9)	Five had other drugs also detected; four also had pregabalin detected	Guerrieri et al. (2017b) [57]
U-47700 death in Texas (USA)	GC-MS	U-47700: blood (f) 0.36 mg/L	Young male found dead with 3-fluorophenmetrazine (3-FPM, Blood (f) = 2.4 mg/L) also detected together with amitriptyline, diazepam, methamphetamine tr, flubromazolam and delorazepam	Ellefsen et al. (2017) [122]
40 acrylfentanyl fatalities in Sweden	LC-MS/MS	Blood 0.01– 5 ng/g (median 0.2 ng/g)	Most had other drugs also detected	Guerrieri et al. (2017) [42]
Fatalities in Germany	LC-QTOF-MS	Case 1: AH-7921, blood (f) 0.45 mg/L; blood (h) 0.48 mg/L; liver 0.53 mg/kg; urine 0.76 mg/L; VH 0.19 mg/L; hair detected Case 2: MT-45, blood (f) 0.66 mg/L; blood (h) 1.3 mg/L; liver 0.024 mg/kg; urine 0.37 mg/L; VH 0.26 mg/L	Drug-caused deaths, primarily by opioid, but other drugs also present Case 1: trace or low amounts of methadone, diphenhydramine, tetrazepam, methamphetamine, mirtazapine Case 2: trace or low amounts of lidocaine, PB-22, 5F-AKB-48	Fels et al. (2017) [72]
Two 4-fluorofentanyl deaths in Germany	LC-MS/MS	4-Flourofentanyl: blood 25–35 ng/mL; also detected in other specimens	Both suicides; young male and female with history of psychological problems and abuse of narcotics	Strehmel et al. (2017) [87]
Two furanylfentanyl deaths in Canada	No details given	Case 1: furanylfentanyl, blood 1.1 ng/mL Case 2: furanylfentanyl, blood 0.68 ng/mL (AM)	Both young women using Perocet but containing this fentanyl and alprazolam. Highlights a series of such deaths also seen in British Columbia	Milroy and Kepron (2017) [58]
o-Flourofentanyl death in Sweden	LC-MS/MS, HR-MS	o-Flourofentanyl: blood 2.4 ng/mL, urine 3.9 ng/mL	Young male who had a few days previously been admitted for an overdose found dead from likely snorting a white powder containing opioid; also alprazolam, clonazepam, diazepam metabolite and THC detected	Helland et al. (2017) [86]
25 deaths from fentanyl or fentanyl analogues (carfentanil, with butryfentanyl, flourobutryfentanyl) in UK	LC-HR-MS	Carfentanil: blood 0.09–4 ng/mL (median 0.3) (*n* = 22), AM 0.021–0.098 ng/mL (*n* = 3) Fentanyl: blood 1–3.1 ng/mL (*n* = 6) Butryfentanyl and 4-butryfentanyl detected with carfentanil positive cases Also alfentanil (*n* = 1) and despropionylfentanyl det (*n* = 1), furanylfentanul (*n* = 2)	All cases other drugs also detected, often morphine and more than one fentanyl	Hikin et al. (2017) [49]
U-47700 fatality in San Diego, California (USA)	GC-MS-SIM	Blood (f) 0.19 mg/L; blood (c) 0.34 mg/L; VH 0.17 mg/L; urine 0.36 mg/L, SC trace	Middle-aged man found unresponsive in bed; known drug user, thought to have snorted drug; dilated left ventricle, congested lungs, some steatosis. Alprazolam, doxylamine, nordiazepam, diphenhydramine, ibuprofen, salicylic acid and THC-acid also detected	McIntyre et al. (2017) [123]
47 Acryl(oyl)fentanyl deaths in Nordic countries; numerous ED admissions	Likely to be LC-MS/MS or HR-MS	No details given of concentrations for fatalities; non-fatal mono-intoxications with serum concentrations ranging from 0.8 to 2.1 ng/mL (*n* = 8)	Also see EMCDDA site [124] and [43]	Ujváry et al. (2017) [44]
355 carfentanil deaths in USA	HR-LC/MS	Blood 0.1–14 ng/mL (median 38 ng/mL)	Many were acute deaths but no details were provided; four were human performance cases with blood concentrations 0.41–1.4 ng/mL; most involved other drugs including fentanyl, heroin and cocaine	Papsun et al. (2017) [54]
10 deaths from various fentanyls in Miami, Florida (USA)	LC-ion trap MS	Six cases with carfentanil, 3 cases of p-fluoroisobutryfentanyl and furanylfentanyl, and one each of acetylfentanyl, β-OH-thiofentanyl. Most had 2 or more fentanyls including fentanyl (*n* = 8)	No quantitative data provided; all cases had multiple drugs contributing to death	Shoff et al. (2017) [105]
Death each from carfentanil and furanylfentanyl in Tampa, Florida (USA)	Likely LC-MS/MS at a reference laboratory [125]	Case 1: carfentanil, blood (h) 1.3 ng/mL; furanylfentanyl, blood (h) 0.34 ng/mL Case 2: carfentanil 0.12 ng/mL	Case 1: young male also with traces of morphine and hydromorphone Case 2: young female with traces of cocaine	Swanson et al. (2017) [55]
Seven deaths with acetylfentanyl, plus 10 deaths with fentanyl in Tampa, Florida (USA)	Immunoassay (fentanyl) plus GC-MS-SIM	Acetylfentanyl: blood (f) 2–600 ng/mL (median 0.31 ng/mL) (*n* = 7) Fentanyl: blood 4–38 ng/mL (median 16) (*n* = 10)	All cases were mixed intoxications including these 2 drugs, heroin or other opiates/opioids and/or other drugs	Pearson et al. (2015) [97]
Nine deaths with furanylfentanyl and two with U-47700 in Tennessee (USA)	HR-MS and LC-MS/MS	Furanylfentanyl: blood (f) 2–42.9 ng/mL (median 6.5 ng/mL) U-47700: blood (f) 189 and 547 ng/mL	All cases were mixed intoxications including other opiates/opioids and/or other drugs	Papsun et al. (2017) [60]
U-47700 fatality in Wichita, Kansas (USA)	GC-MS-SIM	Blood (f) 0.4 ng/mL; blood (h) 0.26 ng/mL; urine 4.6 ng/mL; VH 0.09 ng/mL; liver 0.28 ng/mg	Young male obese drug user with enlarged heart and oedematous, congested lungs; THC Blood (h) 19 ng/mL, possible trace phencyclidine	Rohrig et al. (2017) [82]
Three acrylfentanyl fatalities (one also with furanylfentanyl) in Charleston, South Carolina (USA)	GC-MS-SIM	Acrylfentanyl: blood (p) 0.3, 0.95 and 0.32 ng/mL Furanylfentanyl: blood (p) 0.95 ng/mL (case 3)	Three male drug users, all with other drugs detected. LOQ 0.1 ng/mL	Butler et al. (2017) [46]
Numerous carfentanil deaths from various US states	LC-MS/MS	Carfentanil: blood 10–2 000 pg/mL (median 193 pg/mL) (*n* = 262) 13 case reports listed: blood 10–529 pg/mL (median 114 pg/mL)	Most cases involved other drugs, and in a few heart disease; LOD 5 pg/mL, LLOQ 10 pg/mL	Shanks and Behonick (2017) [125]
4-Fluoroisobutyr-fentanyl fatality in Sweden	LC-MS/MS and HR-MS	4-Fluoroisobutyrfentanyl: serum 38 ng/mL	No other drugs detected; opioid used IV Other opioids detected in other non-fatal admissions to emergency	Helander et al. (2017) [43]
41 acetylfentanyl fatalities in Pennsylvania (USA)	ELISA and GC-MS-SIM	Acetylfentanyl: blood (f,h) 0.1–2 100 ng/mL (median 11 ng/mL)	26 cases were also with fentanyl; all bar one were multiple drug toxicities; mono-intoxication: blood (f) 170 ng/mL	Dwyer et al. (2017) [38]
Furanylfentanyl fatality in San Francisco, California (USA)	ELISA and GC-MS-SIM; confirmation possibly by LC-MS/MS	Blood (p) 1.9 ng/mL; blood (h); VH <0.2 ng/mL, 2.8, SC 55 μg 4-ANPP metabolite also detected	Young man found dead following ingestion of a blue pill resembling oxycodone, found to be furanylfentanyl. No other drugs detected. Pulmonary and cerebral oedema, some coronary artery disease	Martucci et al. (2017) [59]

*Publications arranged in order of publication year; f: femoral; h: heart; s: subclavian; c: central; p:peripheral; VH: vitreous humour; SC: stomach contents; LC-MS/MS: tandem mass spectrometry with liquid chromatography; GC-NPD: gas chromatography with nitrogen phosphorous detection; GC-MS-SIM: selected ion monitoring mass spectrometry with gas chromatography; HR-MS: high-resolution mass spectrometry; PAD: photodiode array detection; STRIDA: Swedish project involving Karolinska Institute and Laboratory and Swedish Poisons Information Centre; AM: antemortem; 4-Methoxy PV8: 1-(4-methoxyphenyl)-2-(pyrrolidine-1-yl)hepatan-1-one; 4-ANPP: 4-anilino-*N*-phenethylpiperidine; ELISA: enzyme linked immunosorbent assay; LC-QTOF-MS: liquid chromatography-quadruple time-of-flight mass spectrometry.

It is metabolized primarily to the inactive norfentanyl (removal of the phenethyl moiety on the piperazine nitrogen) predominately by P450 3A4, and less so to inactive hydroxyfentanyl and hydroxynorfentanyl [24], with the latter two further converted to conjugates [25]. Despropionylfentanyl has also been detected in plasma, but not urine [26].

Numerous publications have described fatalities associated with its misuse [5–7,9–17,27–29]. As with most drug-caused deaths other drugs have also been used and often also misused in combination. Peripheral blood concentrations range from near 1 ng/mL to well over 20 ng/mL with a median somewhere between 5 and 10 ng/mL, depending on degree of tolerance and presence of other significant drugs. Doses vary significantly depending on the route of administration and degree of tolerance [30]. For example, typical carfentanil doses are of the order of several micrograms, compared to fentanyl that range up to about 1 mg and less potent opioids may be up to about 10 mg; however, the dose will also depend on the degree of tolerance to opioids. Intravenous use, smoking and nasal insufflation provide a rapid absorption and pharmacological effect, whereas as oral or intramuscular administration will provide a slower onset of action. A common report was misuse of transdermal patches either by applying multiple patches on the body or injecting the contents of a used patch [31–35].

### Novel opioid fatalities

Table 1 also summarizes reported fatalities of other fentanyl-like opioids and some other novel opioids.

#### Acetylfentanyl

This has been the most common novel opioid with 11 publications with reported deaths from drug toxicity in at least several European countries and across much of the USA. It appears to be threefold less potent than fentanyl itself [36] and death has occurred when used by insufflation as well as intravenously and orally [37]. Blood concentrations in fatalities show a very large range from 0.1 to 2 100 ng/mL, although the median is about 10 ng/mL. Almost all cases are fatalities from multiple drugs. In the three reported mono-intoxication cases the blood concentrations were 170 ng/mL (femoral) [38], 260 ng/mL [39] and 270 ng/mL (heart) [40], respectively.

#### Acrylfentanyl

This fentanyl sometimes known as simply acryloylfentanyl is slightly more potent than fentanyl in displacing labelled naloxone from the mu-opioid receptor (IC50 1.4 nmol/L) [41]. Deaths from this opioid have largely been restricted to Sweden and other Nordic countries with cited publications listing numerous drug-caused deaths with blood concentrations ranging from 0.01 to 5 ng/g with a median blood concentration of about 0.2 ng/g [42–44]. These deaths included evidence of nasal insufflation of both sprays and crushed tablets. This drug was believed to enter Sweden and neighbouring countries of Denmark, Estonia, Finland and Latvia as well as Slovenia as a powder [45]. More recently acrylfentanyl was detected in the deaths of three male drug users in the USA; all of whom had blood concentrations less than 1 ng/mL [46].

#### Butyrfentanyl

Sometimes also termed butyrylfentanyl is less potent than fentanyl (Ki 32 nmol/L cf 1 nmol/L) but has also caused deaths in Europe and the USA. Two case reports report fatalities attributed to this opioid with femoral blood concentrations of 58 and 66 ng/mL [47,48]. In one of these acetylfentanyl was also detected as well as cocaine with levamisole [47]. In another publication this opioid was found in deaths with other fentanyl or fentanyl analogues, such as carfentanil [49]. By comparison, two publications reported survival from use of this opioid [50,51]. Helander et al.[43] reported serum concentrations in the three cases ranging from 0.6 to 66 ng/mL; showing considerable range in concentrations and overlap with concentrations than those found in the fatalities.

#### Carfentanil

This opioid is used primarily as an incapacitating agent for large animals, and it is even more potent than 3-methylfentanyl. It is some 100 times more potent than fentanyl [52] and was reportedly used in combination with another opioid, remifentanil, in the Melnikov street theatre (Moscow) siege of 2002 as an aerosol to subdue terrorists that claimed over 100 lives [53]. A large series (*n* = 355) of carfentanil deaths were reported from the USA with blood concentrations ranging from 0.1 to 14 ng/mL [54]. Two deaths were reported in one case report from Florida, one of which was in combination with furanylfentanyl and gave postmortem heart blood concentrations of 0.12 and 1.3 ng/mL [55]. In another Florida report, two more deaths were reported from this opioid, with blood concentrations of 0.12 and 1.3 ng/mL; one of which (highest concentration of carfentanil) also involved furanylfentanyl [55]. This ultra-potent opioid has also been seen in the UK in which 25 deaths were reported with blood concentrations ranging from 0.09 to 4 ng/mL, often in combination with other fentanyls and morphine [49].

#### Furanylfentanyl

This opioid which is about five times less potent than fentanyl has been reported in deaths in Canada, Sweden and the USA [55–59]. Mohr reported eight cases with furanylfentanyl, of which five were in combination with another opioid, U-47700 [56] while Papsun reported a series of nine deaths in USA from this opioid in combination with other drugs [60]. Blood concentrations in these two series ranged from 2 to almost 76 ng/mL with a median of about 10 ng/mL. In a Swedish series of seven fatalities, blood concentrations ranged from 0.38 to 2.7 ng/mL, again with most using other drugs [57]. Almost all cases had oedematous lungs recorded at autopsy. Four also had pregabalin detected. There was some evidence provided that this fentanyl was reasonably stable *in vitro*. The Canadian case report described two women that died using counterfeit tablets labeled as Perocet; blood concentrations were 0.68 and 1.1 ng/mL [58] and in another case a young man died following ingestion of a blue pill resembling oxycodone [59]. In one case of survival reported by the Swedes, the serum concentration was 148 ng/mL [37].

#### Methylfentanyls

3-Methylfentanyl, known as “China White”, was first reported to cause hospitalizations and many deaths in California (and some in neighbouring States) and Pennsylvania in the 1980s as well as a number of other fentanyl derivatives [20,61]. The more active cis-isomer is about 7 000 times more potent as an opioid as morphine. In 1980s, a death from use of α–methylfentanyl [62] was also reported. The occurrence of methylfentanyls is characterized by relatively short-lived epidemics. This is probably due to the high potency of the drug with low doses and subsequent dilution problems, causing a significant risk of overdosing as well as closure of clandestine laboratories [61].

A series of 16 3-methylfentanyl deaths was also reported from Allegheny County in Pennsylvania [63]. Over a decade ago, there were three deaths reported by use of 3-methylfentanyl in Finland [64] and later an epidemic in neighbouring Estonia [65]. Blood concentrations were often less than 1 ng/mL. In the Estonian series of over 100 fatalities, the cis-3-methylfentanyl blood concentrations ranged up to about 2 ng/mL with a median of about 1 ng/mL [65].

Using LC-MS/MS, it was for the first time possible to determine cis-3-methylfentanyl in the blood of victims of fatal overdose, the mean concentration being 0.5 g/L (range 0.3–0.9 g/L). These values are significantly lower than the levels reported above for α-methylfentanyl and fentanyl. Despite the presence of other drugs, poisoning by 3-methylfentanyl was in each case considered the underlying cause of death. This was due to death appearing to occur immediately following injection of the drug. The victims’ ages, ranged from 30 to 41 years, were higher than those typically found in fatal heroin poisonings in Finland; half of the victims of heroin fatalities have been younger than 25 years.

#### 4-Methoxybutyrfentanyl

Four deaths due in part to 4-methoxybutyrfentanyl were also from the Swedish group, all of whom had low serum concentrations (1.3–11 ng/mL) of this fentanyl but who had other drugs also present [37]. Again, symptoms were suggestive of opioid overdose.

#### Ocfentanil

Two case reports describe deaths of young men using this opioid by nasal insufflation with postmortem femoral blood concentrations of 9.1 and 15 ng/mL [66,67]. In both cases, no other drugs were apparently involved. This opioid is about twice as potent as fentanyl.

#### AH-7921

This opioid was first reported in deaths in Sweden in 2014 in which nine cases were described with blood concentrations ranging from 0.08 to almost 1 mg/L [68]. It has a similar potency on the μ-opioid receptor as morphine. In the same year, a fatality in USA [69] and two deaths in Norway [70] were reported, followed by five deaths in the UK (blood concentrations 0.05 to 4.46 mg/L) [71] and a case report from Germany [72]. While low concentrations can be expected particularly if death is delayed or other significant drugs are operative blood concentrations tend not to be that low with a recorded median of about 0.3–0.4 mg/L. This drug was the subject of a Critical Review Report to the United Nations in 2014 [73] and a review [74].

#### MT-45

This opioid was first detected in Sweden in late 2013 and resulted in 28 deaths from November 2013 to July 2014 leading to a risk assessment conducted by the European Monitoring Centre for Drugs and Drug Addiction (EMCDDA) [75]. It is a disubstituted *N,N*’-piperazine also known as I-C6 and is taken in much the same way as other fentanyl-like opioids (nasal insufflation, oral, smoking) with a potency somewhat higher than morphine. Doses around 15–75 mg are used depending on route and degree of tolerance. In these 28 deaths, almost all were due to drug toxicity with the median blood concentration was 0.35 μg/g and as other opioids most involved use of other drugs. In eight cases, the cause of death was listed as MT-45 intoxication, however, the median was actually higher at 0.8 μg/g (range 0.2–1.9 μg/g). In nine non-fatal intoxications the blood concentrations ranged from 6–157 ng/mL (median 47 ng/mL) [76]. In the peer-reviewed literature a further two publications involving single case reports of young males in which death occurred from use of this opioid in conjunction with other drugs giving blood concentrations of 0.52 and 0.66 mg/L [72,77]. It was also reported as present in illegal products in Japan in 2014 [78].

#### U-47700

This opioid is a structural isomer of AH-7921 (see earlier). While less potent than fentanyl numerous deaths resulting from its use have been reported worldwide including the USA, UK and Belgium. Fatal toxicity was first reported in 2016 in the UK of a young male snorting this drug [79]. In the same year, a death was reported from Belgium of a young man inhaling fumes, in combination with fentanyl [80] and a series of 16 cases from the USA in which five also involved furanylfentanyl [56]. These and seven other publications in 2017 found blood concentrations of U-47700 ranging from 0.4 ng/mL to 1.46 mg/L with a median of around 0.3 mg/L (Table 1). As with other opioids most had other drugs present including the case report from the USA in which the stimulant 3-fluorophenmetrazine was also detected [81], and in another, of a morbidly obese man with an enlarged heart [82]. Survival from use of this opioid has also been reported: all patients showed classical signs of opioid toxicity [83,84]. In one case, serum concentration of this opioid during hospitalization was 394 ng/mL and the desmethylated and hydroxylated metabolites were also detected in urine [84]. In the 2 years to end 2016, the DEA reported at least 46 fatalities linked to the use of U-47700, largely in New York and North Carolina [85].

#### Other novel opioids

4-Fluorobutyrfentanyl was detected in two drug-related fatalities in Poland in a young male and young female. The male at least presumably smoked this drug through an e-cigarette (with nicotine) [6] (Table 1).

A young intravenous drug user was admitted to emergency but died some time later. He was found to have 4-fluoroisobutyrfentanyl with a serum concentration of 38 ng/mL a few hours after admission [43].

Recently two 4-fluorofentanyl and one 2-fluorofentanyl deaths were reported in Germany and Sweden, respectively [86,87]. The 4-fluorofentanyl cases were both suicides, while the Swedish case initially involved two young men who had previously been admitted to hospital following toxicity to snorting 2-fluorofentanyl but recovered after supportive care and treatment for opioid overdose and were subsequently released one day later. Unfortunately, one of these was found deceased a few days later due to drug toxicity, largely from this fentanyl (blood concentration 2.4 ng/mL) in the presence of three benzodiazepines, cannabis and GHB.

More recently, one death was reported from Tennessee (USA) of an anaesthetist who died from self-injection of sufentanil and midazolam [88]. In this case, the heart blood concentration of sufentanil was 1.1 ng/mL.

Other novel opioids based on fentanyl have been identified in emergency hospital admissions that did not lead to death. These were 4-chloroisobutryfentanyl, cyclopentylfentanyl and tetrahydrofuranfentanyl [43].

### Pathology findings

The most common and consistent findings on autopsy of persons that have died from novel opioid toxicity is that seen in heroin and other deaths from opiates, notably heavy lungs associated with pulmonary oedema and hyperaemia, with pneumonia also seen in some cases, particularly in cases where death process was relatively long [42,50,57,62,67,72,89,90]. Often cerebral oedema and congestion in liver and other organs is also noted. This is consistent with death from depression of the central nervous system. In hospital admissions high heart rate, high blood pressure with signs of apnea and miosis is also commonly seen [37].

However, some unusual individual pathology has been observed. MT-45 has been linked to bilateral hearing loss, a side effect also reported elsewhere for this drug [72,77] and in one case a known heroin user developed significant haemoptysis, acute lung injury, hypoxic respiratory failure and diffuse alveolar haemorrhage following use of butyrfentanyl [50]. He survived and was released from hospital after 1 week [50]. Diffuse alveolar haemorrhage was also seen in a intranasal user of fentanyl [91] and is probably a rarer side effect of opioid toxicity [92].

Other pathology is sometimes noted, and may also contribute to death, but the drug does not cause this directly, rather, is present for other reasons, such as coronary artery disease.

### Methods of analysis

Before the widespread use of tandem MS (MS/MS) and/or high-resolution MS (HR-MS) specimens were analysed by immunoassay and if positive by GC-MS with selected ion monitoring (SIM). Immunoassays tend to have a limit of detection from about 0.25 ng/mL to about 2 ng/mL, and have been able to detect the presence of some other fentanyls due to cross-reactivity [93–95]. Depending on the antibody used this includes acetylfentanyl, butyrfentanyl, furanylfentanyl, 4-methylfentanyl, 4-fluorofentanyl, but not alfentanil or carfentanil [94–96]. It is possible, but not confirmed, that related structures involving *N*-alkylated piperazines may also cross-react including risperidone and 9-hydroxyrisperidone (paliperidone) [94].

The Randox biochip platform enables detection of acetylfentanyl, carfentanil, furanylfentanyl, ocfentanil, remifentanil and sufentanil, as well as AH-7921, MT-45 and U-47700 with cut-offs ranging from 0.25 (carfentanil) to 10 ng/mL (U-47700) in urine [personal communication].

Before the widespread availability and use of MS/MS and HR-MS GC-MS, operating in the SIM mode was used to detect fentanyl and even some of the other fentanyls, however, modern GC-MS instruments appear to have sufficient sensitivity to detect sub-nanogram per millilitre concentrations of many of the synthetic opioids, such as those reported in some recent publications [46,82,97].

Fentanyls and related designer opioids are usually well extracted in organic solvents from basified blood/plasma/serum or another liquidified specimen. Butyl chloride has been used for this purpose [31,65], however, isooctane has also been used [17]. Solid phase extraction (SPE) has also been used successfully [33]. Detection limits of fentanyl have ranged from 0.5 to 2 ng/mL and have often also included norfentanyl.

Table 2 summarizes procedures published since 2000 that were validated to detect 3 or more fentanyls. These procedures were also designed to quantify opioids in blood/serum and urine, and sometimes also other specimens that can be collected postmortem. These nine publications were validated to detect three [56,98], five [99], six [100,101], nine [102], 10 [103], 13 [104] and 14 fentanyls [105], respectively. All bar two used SPE and all bar one used either LC-MS/MS or ion trap MS instruments. The one that used GC-MS (operated in SIM mode) used a pentafluorobenzamide derivative that also gave the best limit of detection of 0.002 5 ng/mL (or 2.5 fg/mL). The LOD of other procedures varied from 0.003 to 0.5 ng/mL. The higher grade MS/MS or MS instruments will usually provide a higher level of sensitivity. The desired LOD/LLOQ will depend on the opioid. For example, carfentanil one of the most potent fentanyls will require limits down to at least 0.01 ng/mL to be reasonably able to detect use in cases.

**Table 2.** Analysis details for published methods targeting two or more fentanyls.

Type of Analysis	Specimen(s)	Extraction technique and conditions	Chromatography	Analysis technique and conditions	Reference*
Quantitative analysis of fentanyl, alfentanil, sufentanil and metabolites	Urine (no hydrolysis)	SPE using Extrelut® NT1; elution with *n*-heptane/isoamyl alcohol (98.5:1.5)	GC using pentafluorobenzamide derivatives on a DB-35 capillary column	MS-SIM; LOD from 0.002 5 ng/mL; deuterated IS	Van Nimmen et al. (2004) [98]
Quantitative analysis of 13 fentanyls: alfentanil, carfentanil, fentanyl, lofentanil, ohmefentanyl, 3-methylfentanyl, α-methylfentanyl, sufentanil and some metabolites	Urine (no hydrolysis)	SPE Oasis HLB C_18_ columns; eluted with MeOH	LC using a Xterra MS C_18_ column (2.1 mm × 150 mm, 3.5 μm); ammonium acetate buffer in 95:5 MeOH/ACN gradient	Micromass Quattro Ultima triple quadrupole MS; LOD 0.003 – 0.027 ng/mL; deuterated IS	Wang et al. (2006) [104]
Quantitative analysis of 9 fentanyls: alfentanil, fentanyl, p-fluorofentanyl, cis-3-methylfentanyl, trans-3-methylfentanyl, α-methylfentanyl, norfentanyl, remifentanil, sufentanil and other opioids	Blood, urine (β-glucuronidase hydrolysis) (PM)	LLE extraction using butyl acetate	LC using Gemini C_18_ column (100 mm × 2.0 mm, particle size 3 μm); using an acetonitrile–ammonium acetate gradient at pH 3.2	MS/MS Sciex 3200 QTrap, MRM; LOQ 0.01–0.2 ng/mL; deuterated IS	Gergov et al. (2009) [102]
Quantitative analysis of 6 fentanyls: alfentanil, fentanyl, 3-methylfentanyl, remifentanil, norfentanyl, sufentanil	Plasma and urine	LLE from K_2_CO_3_ basified specimen using *n*-hexane:ethylacetate (7:3)	LC XTerra MS C_18_ (2.1 mm × 150 mm, 3.5 μm); moving phase 0.15% formic acid in ACN gradient	MS/MS with MRM (Waters Quattro) LLOQ from 0.1 ng/mL; deuterated IS	Cooreman et al. (2010) [100]
Quantitative analysis of 10 fentanyls: alfentanil, carfentanil, fentanyl, lofentanil, 3-methylfentanyl, α-methylfentanyl, sufentanil, and some metabolites	Urine (no hydrolysis)	SPE Oasis HLB 30 μm columns; eluting with 1% formic acid in ACN, plus on-line extraction	LC Symbiosis system using a 3.0 mm × 50 mm XTerra MS C_18_ column with 2.5 μm; 1% formic acid ACN gradient	Sciex 5500 Qtrap, ESI, RM; LOQ 0.01 – 0.05 ng/mL; deuterated IS	Shaner et al. (2014) [103]
Quantitative analysis of 5 fentanyls: alfentanil, fentanyl, norfentanyl, remifentanil, sufentanil and other opioids	Serum/blood and PM tissues	SPE with Bakerbond C_18_; eluted with DCM/isopropanol/ammonium hydroxide (40:10:2)	LC using Zorbax Eclipse Plus C_18_ (2.1 mm × 150 mm, 1.8 μm); eluted with DCM/2-propanol/ammonium hydroxide (40:10:2)	MS (Agilent 6490 TQ) ESI MRM; LLOQ 0.1 ng/mL or higher; deuterated IS	Eckart et al. (2015) [99]
Quantitative analysis of furanylfentanyl, U-47700 and U-50488	Blood	SPE using CleanScreen® DAU columns; elution with® DCM/MeOH/ammonium hydroxide (78:20:2)	LC using Zorbax Eclipse plus C_18_ (4.6 mm × 200 mm, 3.5 μm); mobile phase 0.1% formic acid in MeOH	MS/MS MRM (Agilent TQ); LOD 0.5 ng/mL, LOQ 1 ng/mL; deuterated IS	Mohr et al. (2016) [56]
Quantitative analysis of 6 fentanyls: acetylfentanyl, carfentanil, 3-methylfentanyl, 2-furanylfentanyl, norfentanyl; fentanyl	Blood, vitreous humour	SPE using mixed mode CleanScreen® ZSDAU020 cartridges; then eluted with DCM:isopropanol:ammonium hydroxide (78:20:2)	LC Kinetex F5 column (50 mm × 2.1 mm I.D., particle size 1.7 μm); 0.1% formic acid and ACN gradient	Thermo ESI MRM LC-MS/MS LOQ from 0.1 ng/mL acetylfentanyl, carfentanil; deuterated IS	Sofalvi et al. (2017) [101]
Qualitative analysis for 15 fentanyls (alfentanil, 3-methylfentanyl, acetylfentanyl, β-hydroxyfentanyl, Butyrfentanyl, carfentanil, desproprionylfentanyl, fentanyl, norfentanyl, furanylfentanyl, p-fluorobutyrfentanil, p-fluoroisobutyrfentanyl, sulfentanil, U-47700, W-18 and 30 other opioids/analgesics)	Blood, urine, liver and brain homogenates	SPE CleanScreen® mixed mode from basified specimen; washed, then eluted with DCM:isopropanol:ammonium hydroxide (78:20:2)	uHPLC (Thermo Acclaim RSLC 120 C_18_ – 2.1 mm × 100 mm, 120 A); mobile phase 2 mmol/L ammonium formate, 0.1% formic acid, ACN gradient	Ion Trap-MS (Bruker AmaZon Speed) ESI full scan with MS^2^ and MS^3^ for selected compounds; LOD 0.1-0.5 ng/mL; deuterated IS	Shoff et al. (2017) [105]

*In order of publication starting 2000; A: angstrom; ACN: acetonitrile; DCM: dichloromethane; DAU: drugs of abuse; ESI: electrospray ionization; IS: internal standard; LC: liquid chromatography; LLE: liquid–liquid extraction; LOD: limit of detection; LOQ: limit of quantitation; LLOQ: lower limit of quantitation; GC: gas chromatographic; MeOH: methanol; MRM: multiple reaction mode; MS: mass spectrometry; PM: postmortem; SPE: solid phase extract.

## Discussion

Since the beginning of the 1990s, a large number of reports on the abuse of fentanyl analogues appeared in the scientific literature, with numerous reported deaths. This occurrence may, in part, have been due to the temporary reduction in the availability of heroin decreasing drastically during the Afghanistan crisis. Some of these were summarized in Table 1 with the majority occurring in the USA and Western Europe [8,10,28,31,33,68,80,106]. Initially these were caused by abuse or misuse of fentanyl by practitioners with access to legal supplies of the drug, however, more recently the opioid has become more widely available, largely through skin patches for transdermal absorption [31,33] and also more recently possible deliberate doping of heroin with a fentanyl [10,19,28]. Clandestine manufacture of fentanyl is known and contributes to the availability and misuse of this opioid [107].

Simultaneously, fatalities due to prescription opioids began to rise over the last 2 decades, with alarming mortality, particularly from oxycodone, although other legal opioids have contributed [108–110].

In most of these cases, as it is in most drug-caused deaths, other contributing drugs are detected alongside fentanyl. These are often another opiate or opioid, alcohol, amphetamines, cocaine or one or more of the benzodiazepines. Due to the presence of other drugs, the degree of tolerance to opioids and sometimes the presence of significant natural disease there are no clearly defined minimum fatal concentration of fentanyl with concentrations contributing or causing death from as low as about 0.2 ng/mL, although the median peripheral blood concentrations have tended to be about 10–20 ng/mL [28,111].

The first reports of other fentanyl derivatives causing death was in the 1980's by α-methylfentanyl in California [62] and later from 3-methylfentanyl, or China White, as it was known in which 16 deaths were reported from Allegheny county, Pennsylvania [63]. It was not until about 10 years ago when fentanyl derivatives causing death began to appear on a more regular basis, starting with clusters of 3-methylfentanyl deaths in Finland and Estonia [64,65], and then acetylfentanyl fatalities in the USA a few years later [112].

In 2002, fentanyls gained notoriety in connection with the Moscow Dubrovka theater siege, when the Russian military used a knockout gas to incapacitate Chechen rebels, leading to the loss of more than 100 rebels and hostages. The available evidence suggests that a combination of an aerosolized fentanyl derivative, such as carfentanil, and an inhalation anesthetic, such as halothane, was used [113], although it seems that remifentanil may also have been present in the aerosol [53].

In the last few years, numerous deaths have been reported from 16 novel opioids in various publications including acetylfentanyl, acrylfentanyl, butr(yl)fentanyl, carfentanil, 2- and 4-fluorofentanyls, 4-fluorobutyrfentanyl, 4-fluoroisobutyrfentanyl, furanylfentanyl, α- and 3-methylfentanyls, 4-methoxyfentanyl, ocfentanil, as well as AH-7921, U-47700 and MT-45, reports of which have been summarized in Table 1.

While the concentrations detected bear some relation to the potency of the drug to the opioid receptor(s) with the weaker agonists having concentrations well above the nanogram per millilitre level (e.g. acetylfentanyl, butyrfentanyl and furanylfentanyl) many are substantially more potent than fentanyl and require detection limits well below 1 ng/mL. For example, the most potent of the opioids listed here, carfentanil, in a recent publication was able to be detected down as low as 5 pg/mL (5 ng/L) with many cases positive at near this concentration. Only the best MS instruments would be able to detect (and provide sufficient number of confirmatory ions or ion transitions) at such low concentrations in biological matrices.

Unfortunately, on review of the publications listed here (reports of deaths from use of a novel opioid), there is no concentration that could be considered a minimum that can cause death. This is not surprising since there is no such minimum fatal concentration for other opioids, such as morphine (including from use of heroin), methadone and oxycodone. The use of other significant drugs, such as other opiates or opioids, and other illicit drugs, as well as alcohol and benzodiazepines to list some, will also contribute to toxicity. Moreover, tolerance is a substantial factor, or lack of it in some cases, that cannot be assessed from the often scant history obtained in a routine death investigation. Additionally, the route and consequently the rate of administration into the body (mostly to brain stem) and posture if a collapse occurs will often also be a factor that can mean the difference between life and death.

Given that fentanyls and probably most, if not all, the related opioids are lipid soluble and can penetrate tissues much better than the water-soluble morphine there is surprisingly little difference in concentration whether blood was drawn from the heart or a peripheral site (e.g. femoral, iliac, sub-clavian). Often drugs with high lipid solubility have higher tissue concentrations leading to diffusion into pooled blood giving give to redistribution phenomenon postmortem [114]. The lack of concentration changes postmortem may be due to tight binding of these drugs to tissue structures. In the 16 examples provided in Table 1 where there were paired peripheral and central blood quantitative results the median ratio of central to peripheral blood concentration was 1.3 and only nine of the cases displayed a higher concentration in the central site (range of ratios were 0.4–3.1). Other research has found increases in fentanyl concentrations in blood over one day from pre-autopsy to autopsy [115], which is not surprising given that the drug will have higher concentrations in tissues surrounding blood allowing diffusion into pooled blood after death. More generally, there can be significant variation in the quality of blood taken from a deceased person even when no putrefactive processes are evident.

Nevertheless, given the wide variability in concentrations producing a toxic response relative small artefactual changes in concentration are not likely to allow any concentration (other than perhaps extremely high from intentional misuse) to be used as a predictor of toxicity without recourse to the context of the case and other relevant findings including the pathology.

Of vital importance in any death investigation is the overall reliance on the testing laboratory to be able to detect novel opioids, and indeed other potentially toxic substances. For this reason alone, and the knowledge that there are so many toxic substances available to the wider community, a laboratory must be able to detect unknown substances at very low concentrations, rather than just relying on targeted detections, particularly for cases in which the circumstances suggest another substances may have been used, or the cause of a possible drug-caused death is equivocal.

In conclusion, there have been at least 16 novel opioids reported in death investigations and a number more identified in admissions to emergency departments that also have the potential to cause death, most of which are related structurally to fentanyl. Laboratories engaged in identifying poisons will need to beware of these drugs as well as other novel psychoactive substances (NPS) and of course the more widely available illicit drugs and prescription drugs that are encountered in the community.

## Compliance with ethical standards

This paper does not contain any studies with human participants or animals performed by any of authors.
